# Vitamin B12 deficiency in long-term metformin use and clinician awareness: a scoping review

**DOI:** 10.1136/bmjopen-2025-113829

**Published:** 2026-04-20

**Authors:** Ian Parsonage, David Wainwright, Julian Barratt

**Affiliations:** 1 School of Health Sciences, University of Bath, Bath, UK; 2 Nurse Partner, Compass House Medical Centres, Brixham, England, UK; 3 School of Psychology, Health and Clinical Sciences, Aston University, Birmingham, England, UK

**Keywords:** Knowledge, Awareness, Diabetes Mellitus, Type 2

## Abstract

**Abstract:**

**Background:**

Metformin is the first-line treatment for type 2 diabetes mellitus (T2DM). Long-term use of metformin has been associated with vitamin B_12_ deficiency, which may lead to serious complications such as anaemia and neuropathy. Although international bodies have recommended screening for vitamin B_12_ deficiency in patients on long-term metformin, it is unclear how aware clinicians are of this adverse effect and to what extent such guidance is being followed in practice.

**Methods:**

A scoping review was conducted using Joanna Briggs Institute (JBI) methodology. Databases searched included MEDLINE, Medical Literature Analysis and Retrieval System Online (PubMed), British Nursing Index (BNI), Google Scholar, Cochrane, Embase, Web of Science and CINAHL, Cumulative Index to Nursing and Allied Health Literature (EBSCO) alongside searching for grey literature such as EThOS (Electronic Theses Online Service), DART (Digital Access to Research Theses) European and Kings College London Research Portal. Studies published in English from 1990 onwards were included if they addressed clinician awareness or screening practices. Data were extracted and summarised using a structured tool, with themes mapped visually. The literature search was conducted between 1 August 2025 and 1 November 2025 and included studies published from January 1990 onwards.

**Results:**

23 sources were included in the review. 7 studies directly assessed clinician awareness of metformin-associated vitamin B_12_ deficiency, all conducted outside the UK. Across 15 studies reporting screening practices, routine vitamin B_12_ monitoring was uncommon, with annual testing rates in general below 20% of eligible patients (range 2.6%–19.8%). In a large retrospective cohort study of patients on long-term metformin, 44.9% underwent vitamin B_12_ testing, with a mean delay of 990 days from treatment initiation. Screening was predominantly symptom-triggered rather than preventive, and older adults and other high-risk groups were consistently less likely to be tested. Reported barriers included lack of clinical prompts, competing priorities and testing costs.

**Conclusions:**

Clinician awareness of the link between long-term metformin use and vitamin B_12_ deficiency is present but inconsistently translated into practice. Screening practices remain suboptimal despite recent guideline updates. Interventions, such as checklists, prompts and updated training, may support improved adherence. However, no UK-based studies were identified, highlighting a gap in national evidence. Routine, risk-based screening in primary care could prevent significant morbidity associated with undiagnosed vitamin B_12_ deficiency in this population.

**STRENGTHS AND LIMITATIONS OF THIS STUDY**

- Comprehensive multi-database and grey literature search strategy.
- Multiple reviewers contributed to title and abstract screening, but full-text screening was conducted by a single reviewer, which may increase the risk of selection bias.
- No critical appraisal is planned, consistent with scoping methodology.
- Restriction to English-language studies may omit relevant evidence.

## Introduction

 Diabetes mellitus (DM) is one of the most prevalent health issues in the world. Type 2 diabetes mellitus (T2DM) is the most common.^1^ According to the last report of the International Diabetes Federation (IDF), the prevalence of T2DM in adults worldwide in 2021 is 10.8% (537 million people).^2^ In the UK, metformin is the recommended first-line therapy for the treatment of T2DM in patients with normal renal function and is widely used,^3^ with approximately 24.1 million items dispensed in the UK.^4^

The biguanides, a group of drugs comprising metformin and phenformin, were introduced in 1957 as oral antihyperglycaemic agents to treat T2DM.^5^ Metformin has been used for over 60 years to treat type 2 diabetes at the early stages because of its outstanding ability to decrease plasma glucose levels.^6^ Over time, different uses of metformin were discovered, and the benefits of metformin for various diseases and even ageing were verified.^6^

Nonetheless, as with most drugs, there is the potential for side effects. The most prominent side effect for metformin is gastrointestinal-related, which can occur in about 20%–30% of patients and can include abdominal pain, bloating, diarrhoea, nausea and vomiting.^7^ Additionally, up to 10% of patients taking metformin can suffer from malabsorption of vitamin B_12_ leading to a deficiency.^5^ This is thought to be caused by the metformin interfering with calcium-dependent absorption of the vitamin in the terminal ileum.^5^

Vitamin B_12_, or cobalamin, is an essential water-soluble vitamin that plays a critical role in various cellular processes, particularly in DNA synthesis and metabolism.^8^ Vitamin B_12_ is necessary for the development of red blood cells, growth and nervous system maintenance.^9^ The only dietary sources of vitamin B_12_ are animal products, such as eggs, fish and meats.^9^ Vitamin B_12_ is absorbed in the terminal ileum.^10^

Vitamin B_12_ deficiency can cause serious clinical symptoms such as megaloblastic anaemia, paralysis, dementia, fatigue and mood disturbance. If left untreated, serious neurological and neuropsychiatric complications can occur.^11^

The diagnosis of vitamin B_12_ deficiency is based mainly on blood measurements of serum vitamin B_12_ level less than 200 pg/mL.^12^ Nonetheless, to date, uncertainty persists regarding the interpretation of vitamin B_12_ results in clinical practice, particularly due to variability between laboratory assays and the application of diagnostic thresholds.^12^ Indeed, there is still a significant debate within the scientific community about the specific cut-off values that should be applied to define a low vitamin B_12_.^13^

Although several guidelines, including UK NICE (National Institute for Health and Care Excellence) guidance^14^ for vitamin B_12_ deficiency in adults, recommend diagnostic thresholds and recognise long-term metformin use as a well-established risk factor, uncertainty persists in clinical practice regarding the interpretation of vitamin B_12_ results. This uncertainty relates less to the absence of guidance and more to variability between laboratory assays used to measure total vitamin B_12_.

Evidence suggests that different analytical methods show poor agreement, meaning that a single universal cut-off value may not be reliable across laboratories.^15^ Recent work on assay harmonisation highlights that results obtained from the same patient may differ depending on the assay used, with implications for both diagnosis and clinical decision-making.^15^ Clinicians may therefore face uncertainty when interpreting results, particularly when values fall close to commonly cited thresholds.

A relationship between long-term metformin use and vitamin B_12_ deficiency has been long discussed.^16^ As far back as the 1970s, there were case reports in the literature of B_12_ deficiency associated with metformin use, with several observational studies reporting the same, but very little in the way of high-quality, well-powered, prospective studies.^16^ Nonetheless, prior to 2022, there was no official guidance in the UK on management or monitoring of vitamin B_12_ deficiency related to metformin.^16^

One study by Infante^8^ established that there were no guidelines currently available for vitamin B_12_ deficiency screening in patients on metformin therapy, and vitamin B_12_ deficiency remains frequently unrecognised. Therefore, Infante^8^ wrote a ‘field of vision’ article which proposed a list of criteria for a cost-effective vitamin B_12_ deficiency screening in metformin-treated patients, which could serve as a practical guide for identifying individuals at high risk for this condition.^8^

Infante’s^8^ paper concluded that initial screening and subsequent intermittent periodic testing of vitamin B_12_ status in selected patients treated with metformin may be cost effective and should be considered in order to promptly identify and correct vitamin B_12_ deficiency.

The Medicines and Healthcare products Regulatory Agency (MHRA)^17^ in 2022 released a safety alert regarding the monitoring of vitamin B_12_ deficiency while on metformin therapy. The MHRA^17^ recommends checking vitamin B_12_ levels in people treated with metformin who have symptoms suggestive of vitamin B_12_ deficiency.

Furthermore, the MHRA^17^ recommends checking vitamin B_12_ levels as part of annual review bloods, in groups of patients with risk factors for vitamin B_12_ deficiency, who are taking metformin, especially over the long term—this is a significant change to current typical practice.

Prior to the MHRA alert,^17^ there was no guidance issued on this matter.^16^ Currently, in the UK, the T2DM Clinical Knowledge Summary (CKS)^18^ lists vitamin B_12_ deficiency as an adverse effect of taking metformin.

Nevertheless, despite efforts to promote evidence-based practice, there is often still a gap in the translation of research findings into policies and then into clinical practice.^19^ The National Institute for Health and Care Research (NIHR) agrees with this point and argues when such interventions are implemented (or put into practice), the process is often challenging, unpredictable and typically slow.^20^

Awareness of monitoring for adverse effects from medication has been shown to be poor in other similar cases. An example of this is a mixed method study by Speirs^21^ exploring the monitoring of long-term adverse side effects for nitrofurantoin. This study concluded that many clinicians were unaware of the potential for complications. Sarkies^22^ published a paper exploring the gaps between evidence and practice in the care of familial hypercholesterolaemia and identified that further work is required to translate the evidence into practice.

Glasziou and Haynes^23^ report similar findings and highlight how individual clinicians find it difficult to be aware of all the relevant, valid evidence, especially with many important practice changes linked to low-cost pharmaceuticals or non-pharmaceuticals.

For the purposes of this review, clinician awareness is understood to encompass recognition of metformin-associated vitamin B_12_ deficiency, understanding of its clinical relevance and knowledge of recommended approaches to monitoring.

Despite increasing recognition of vitamin B_12_ deficiency as an adverse effect of long-term metformin use, uncertainty remains regarding clinician awareness, routine screening practices and the extent to which existing evidence has been implemented in clinical practice. No previous review has comprehensively mapped this evidence across awareness, screening and implementation. This scoping review therefore aims to map the existing evidence across awareness, screening and implementation.

### Objectives

This scoping review is guided by a primary objective: to explore how awareness of vitamin B_12_ deficiency in patients on long-term metformin is understood, identified and translated into clinical practice by healthcare professionals. The review will address this through three connected subquestions, which will be analysed both individually and collectively to identify common themes and gaps in knowledge translation:

1. Clinician awareness—what is the extent of evidence regarding clinician awareness of vitamin B_12_ deficiency as a side effect of long-term metformin use in patients with type 2 diabetes?
2. Screening and monitoring practices—what are the existing screening and monitoring practices in primary care settings for early detection of vitamin B_12_ deficiency in patients on long-term metformin?
3. Implementation into clinical practice—how is the evidence on clinician awareness and screening practices implemented in frontline clinical practice for patients with type 2 diabetes on long-term metformin?

## Methods

### Protocol and registration

The scoping review protocol has been published in BMJ Open and underwent external open peer review.^24^

### Design

The methods follow the Joanna Briggs Institute (JBI) recommended structure of: Search strategy; Study/Source of Evidence selection; Data Extraction; Data Analysis and Presentation.^25^ The JBI guidance was most recently updated in 2020, and now clearly reflects a relationship with the recent and complementary Preferred Reporting Items for Systematic Reviews and Meta-Analyses extension for Scoping Reviews (PRISMA-ScR).^26^

Scoping reviews are now seen as a valid approach in those circumstances where systematic reviews are unable to meet the necessary objectives or requirements of knowledge users.^27^ There now exists clear guidance regarding the definition of scoping reviews, how to conduct scoping reviews and the steps involved in the scoping review process.^27^ A scoping review may be applicable where a problem cannot be expressed in a single or precise question and where the aim is to identify characteristics/concepts in papers or studies, and to map, report or discuss these characteristics/concepts. In these cases, a scoping review is the better choice.^28^

Systematic reviews exist for each component of the title (vitamin B_12_ deficiency in patients on long-term metformin, and clinician awareness of evidence-based practice), but no previous scoping reviews or studies have combined the two to look at clinician awareness of vitamin B_12_ deficiency in patients on long-term metformin use.

A scoping review was preferable to a systematic review for the question posed because the question covers several concepts and themes in which individual studies and reviews exist, but no mapping has taken place exploring these themes together.

The JBI Manual for Evidence Synthesis, Chapter 11: Scoping Reviews^28 29^ offers a structured framework for enacting and presenting scoping review.^25^

### Information sources

#### Eligibility criteria

Only studies published in English were included. The review was limited to English-language publications due to resource constraints and because the review aimed to inform UK clinical practice, where guidance and implementation strategies are predominantly reported in English. Table 1 shows the inclusion/exclusion criteria. The inclusion and exclusion were as comprehensive as possible within the time and resource constraints of a doctoral study.

**Table 1:** Inclusion/exclusion criteria

Component	Inclusion criteria	Exclusion criteria
Population	Clinicians involved in the care of adults with type 2 diabetes mellitus or prescribing metformin	Studies not involving clinicians
Concept	Clinician awareness of metformin-associated vitamin B12 deficiency and/or screening or monitoring practices	Studies not addressing clinician awareness or screening practices
Context	Primary or secondary care settings	Non-clinical or unrelated settings
Publication date	Published from 1990 onwards	Published prior to 1990
Language	English	Non-English

### Search strategy

Arksey and O'Malley^30^ suggest that a wide definition of key words for search terms should be adopted to glean a ‘broad coverage’ of available literature. The search strategy aimed to locate both published and unpublished studies.

The scoping review considered both qualitative and quantitative studies, including experimental and quasi-experimental study designs, including randomised controlled trials, non-randomised controlled trials, before and after studies and interrupted time-series studies. In addition, analytical observational studies including prospective and retrospective cohort studies, case-control studies and analytical cross-sectional studies will be considered for inclusion. This review also considers descriptive observational study designs including case series, individual case reports and descriptive cross-sectional studies for inclusion.

In addition, systematic reviews that met the inclusion criteria were also considered, depending on their respective research questions. Table 2 shows the search terms used in the search strategy.

**Table 2:** Search terms used in searches

Concept	Search terms (combined using Boolean operators)
Condition/exposure	Vitamin B12; metformin; type 2 diabetes mellitus
Outcome	Deficiency; screening; early detection
Professional group	Clinician; healthcare professional; doctor; nurse; pharmacist
Knowledge construct	Knowledge; awareness
Limits	English language

The databases searched included MEDLINE (PubMed), British Nursing Index (BNI), Google Scholar, Cochrane, Embase, Web of Science and CINAHL (EBSCO), alongside searching for grey literature such as EThOS, DART European and Kings College London Research Portal.

The literature search was conducted between 1 August 2025 and 1 November 2025 and included studies published from January 1990 onwards. The full electronic search strategies for all databases, including filters and limits applied, are provided in online supplemental file 1: Full electronic search strategies.

### Study selection

Following the search, all identified citations were collated and uploaded into EndNote 21.^31^ All retrieved citations were imported into EndNote 21,^31^ where automated and manual de-duplication was performed prior to screening. Titles and abstracts were independently screened by IP, DW and JB against the inclusion criteria. Full-text screening was undertaken by IP. While the involvement of an additional independent reviewer may have further reduced the risk of selection bias, predefined eligibility criteria were applied consistently across all studies. Any uncertainties or disagreements during screening were resolved through discussion with DW and JB until consensus was reached.

**Figure 1:**
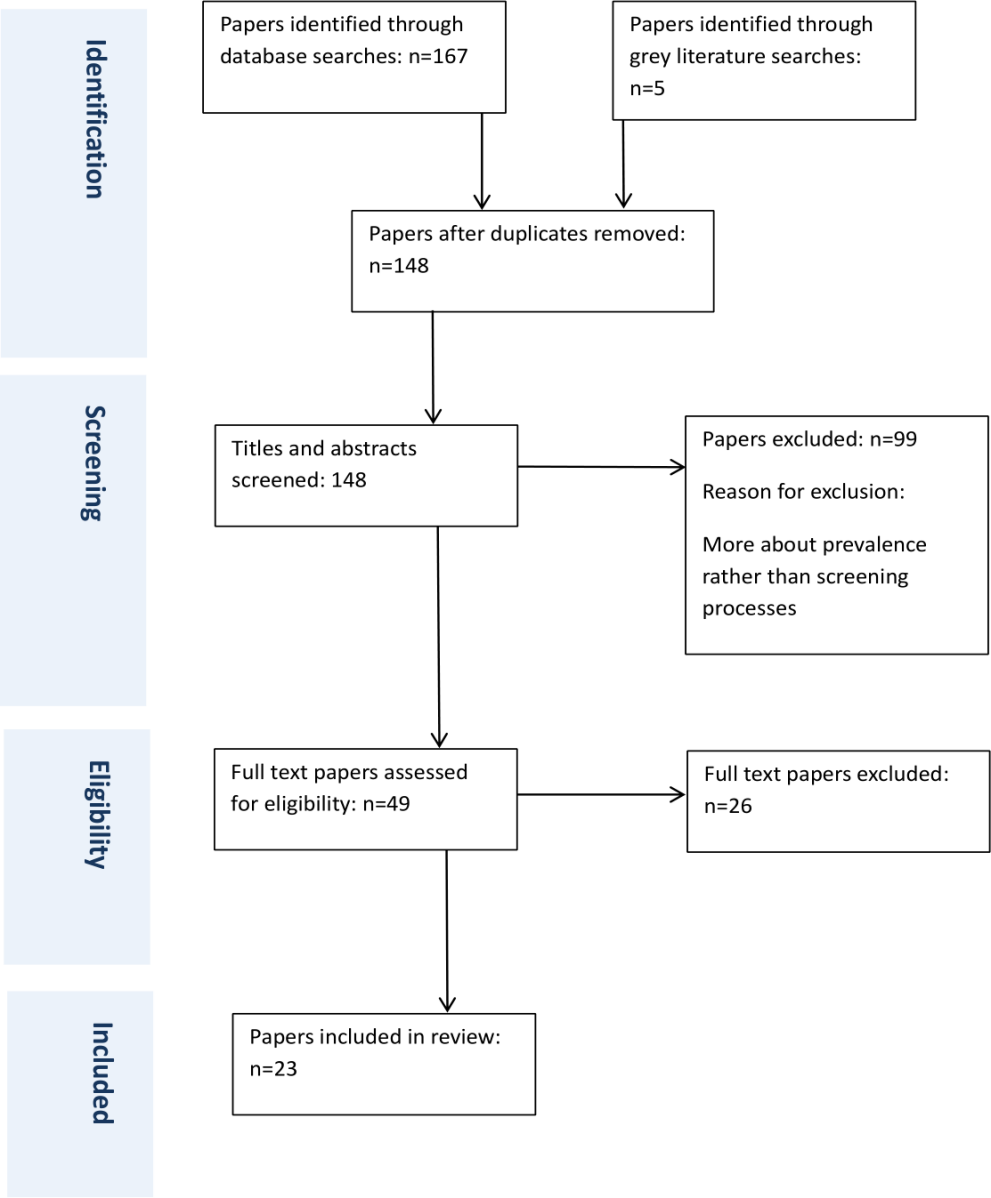
Flow diagram of search.

The full text of selected citations was assessed in detail against the inclusion criteria. Articles were excluded if they did not pertain to awareness or screening of vitamin B_12_ deficiency in type 2 diabetes or metformin use. The results of the search and the study inclusion process are reported in full in the final scoping review and presented in a PRISMA flow diagram (figure 1).^32^

### Data extraction

Data were extracted from papers included in the scoping review by the reviewer using a data extraction chart developed by the lead author and based on the JBI data tool.^26^ This sheet includes information about the name of study, author, year of publication, origin/country, aims/purpose, population and sample size, methodology/methods, outcomes and details of these key findings that relate to scoping review. For extracting data from the discussion papers, the emphasis was placed on extracting apparent themes from the papers and then interpreting those themes using clinical and methodological judgement.

## Results

### Study selection

The database searches identified 23 sources that met the inclusion criteria following de-duplication and full-text screening. The study selection process is presented in the PRISMA flow diagram (figure 1). Consistent with scoping review methodology, no formal critical appraisal of included studies was undertaken. The completed data extraction tool can be found in online supplemental file 2: Full data extraction table.

### Synthesis of findings

**Figure 2:**
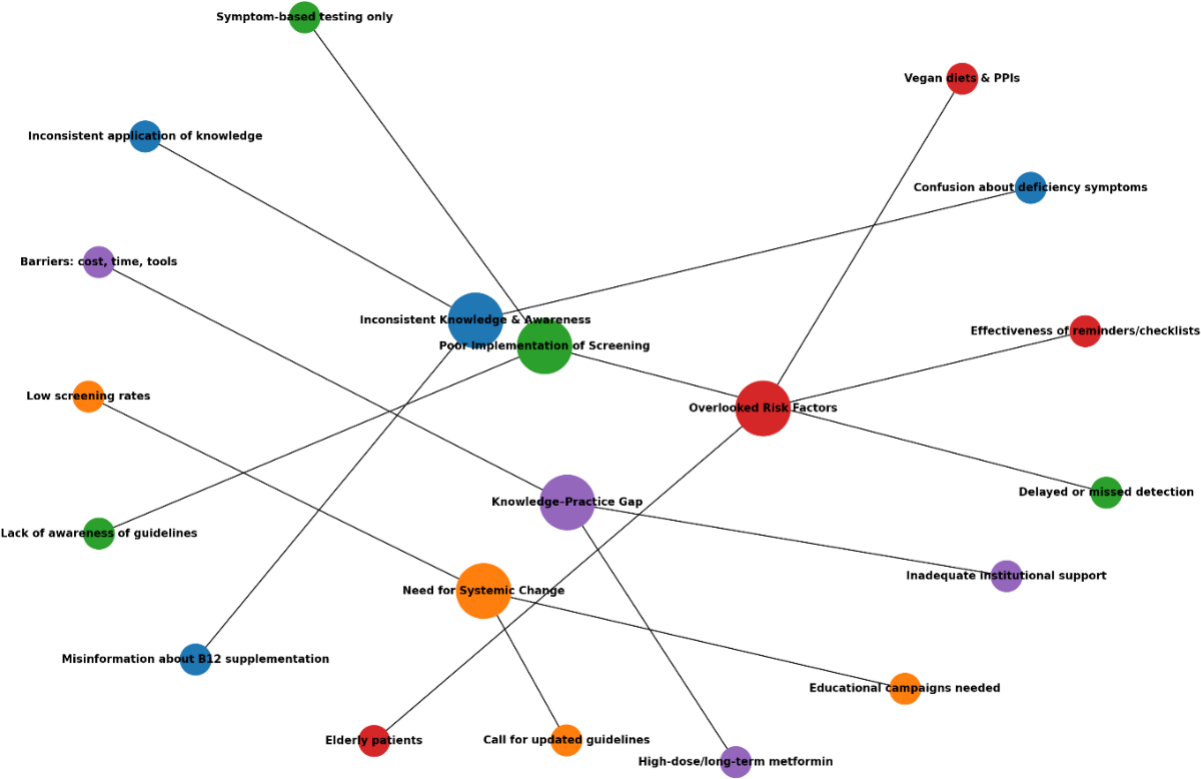
A visual map of theme generated from the analysis of the data extracted.

Figure 2 presents a visual mapping of key themes identified across the included studies, generated using weighted frequencies derived from the data extraction table. The dominant themes related to clinician awareness, variability in screening practices and barriers to implementation.

The key characteristics of included sources and their contributions to each review objective are summarised in table 3.

**Table 3:** Summary of data extracted in scoping review.

Review objective	Studies contributing	Geographical coverage	Summary of key findings
Clinician awareness of metformin-associated vitamin B_12_ deficiency	Direct awareness studies: Alwadi,^1^ Aldossari,^36^ AlSaad,^37^ Alshammari^38^ Alhaji,^43^ Bhanja38, Martin^34^	Saudi Arabia, Jordan	Across studies, clinicians generally recognised the possibility of B_12_ deficiency with metformin, but detailed awareness was inconsistent and incomplete. Several studies showed clinicians misunderstood diagnostic cut-off values, underestimated risk severity or were unaware of American Diabetes Association (ADA) guidance. Indirect evidence from large database studies suggested a system-level under-recognition, as testing rates remained low despite increased evidence.
Screening and monitoring practices in long-term metformin users	American Diabetes Association (ADA),^39^ Filioussi,^5^ Fogelman,^47^ Davies,^16^ Ghiya,^40^ Herbert,^41^ Longo,^48^ Martin,^34^ May,^42^ Ramzan,^33^ MHRA,^17^ Shivaprasad,^44^ Trout,^49^ Holt,^50^ Kibirige,^51^ Peacok,^52^ Wee^46^	USA, Saudi Arabia, Jordan, Greece, Singapore	Across all studies, screening practices were suboptimal, with most showing routine monitoring occurring in only 2%–20% of eligible patients. Testing was typically symptom-driven rather than preventive. Delays of several years between metformin initiation and first B_12_ test were common (eg, Martin^34^ showed a mean delay of 990 days). High-risk groups (older adults, high-dose metformin users, vegans, polypharmacy) were least likely to be tested. Despite guideline evolution (ADA,^39^ MHRA),^17^ studies consistently reported poor alignment between recommended and actual screening behaviour.
Implementation of evidence into routine clinical practice	Alwadi,^1^ Ghiya,^40^ Herbert,^41^ May,^42^ MHRA^17^	USA, Middle East	Implementation studies demonstrated a clear knowledge–practice gap. Barriers included lack of prompts, high testing costs, patient refusal, clinician workload and competing priorities. Checklists/prompts (eg, Herbert)^41^ showed marked improvements in B_12_ testing, indicating that simple structural changes can shift practice patterns. MHRA’s^17^ 2022 alert represents a major policy level driver, but no study has evaluated implementation within UK primary care, highlighting a critical evidence gap.

MHRA, Medicines and Healthcare products Regulatory Agency.

Figure 2 provides a visual summary of the key themes identified across the included studies, originated from weighted frequencies within the data extraction. The most prominent themes related to clinician awareness of metformin-associated vitamin B_12_ deficiency, variation in screening and monitoring practices, and challenges associated with implementing routine monitoring in clinical practice. Less commonly represented themes included uncertainty around diagnostic thresholds, a reliance on symptom-triggered testing, and the role of system-level factors such as electronic prompts and structured review processes.

### Characteristics of included studies

The 23 included sources comprised cross-sectional surveys, retrospective cohort studies, quality improvement projects, guideline documents and narrative reviews. Studies were conducted predominantly in the USA and Middle Eastern countries. No UK-based empirical studies were identified.

### Objective 1: clinician awareness of metformin-associated vitamin B_12_ deficiency

Seven studies directly assessed clinician awareness of vitamin B_12_ deficiency as an adverse effect of long-term metformin use. All were conducted in Middle Eastern healthcare settings. Across these studies, awareness of the association between metformin use and vitamin B_12_ deficiency was high; however, detailed knowledge was variable. Although up to 94% of clinicians recognised the association,^33^ fewer were able to identify diagnostic thresholds or recommended monitoring practices. In one survey, only 43% of physicians reported awareness of the need for routine vitamin B_12_ testing in patients receiving long-term metformin.^1^

Several additional studies did not directly assess clinician awareness but provided indirect evidence of under-recognition, reflected by persistently low rates of vitamin B_12_ testing in routine clinical practice.

### Objective 2: screening and monitoring practices

15 reported data on screening or monitoring practices for vitamin B_12_ deficiency in patients treated with metformin. Across these studies, routine screening was uncommon. Annual testing rates were consistently below 20% of eligible patients, with reported values ranging from 2.6% to 19.8%.

Large retrospective cohort studies demonstrated prolonged delays between metformin initiation and vitamin B_12_ testing. In one study, fewer than half of patients receiving long-term metformin underwent vitamin B_12_ testing (44.9%), with a mean delay of 990 days^34^ from treatment initiation. Among those subsequently identified as deficient, delays exceeding 5 years were reported.

Screening was most frequently initiated in response to symptoms rather than undertaken as part of preventative or routine monitoring. Older adults, patients receiving higher cumulative doses of metformin, and those with additional dietary or pharmacological risk factors were among the least likely to be tested, despite having a higher risk of deficiency.

### Objective 3: implementation of evidence into clinical practice

Six studies reported data relating to the implementation of vitamin B_12_ monitoring into routine clinical practice. Reported barriers included lack of electronic prompts, competing clinical priorities, clinician workload and testing costs. Where interventions such as checklist prompts or structured review templates were introduced, marked increases in vitamin B_12_ testing rates were observed.

Policy-level guidance documents, including professional society recommendations and national safety alerts, were cited as potential drivers for improved practice. However, no studies evaluated implementation following the 2022 MHRA Drug Safety Update^17^ within UK primary care settings.

## Discussion

The findings from this scoping review highlight several recurring themes related to awareness, screening practices and management of vitamin B_12_ deficiency in patients with T2DM who are prescribed long-term metformin.

Objective 1—what is the extent of evidence regarding clinician awareness of vitamin B_12_ deficiency as a side effect of long-term metformin use in patients with type 2 diabetes?

Clinician awareness of vitamin B_12_ deficiency as a recognised adverse effect of long-term metformin use is a necessary prerequisite for effective screening and monitoring in routine practice. However, awareness alone does not necessarily translate into consistent clinical action.^35^

Analysis of the studies demonstrated a recurring theme of inconsistent and occasionally inadequate awareness among clinicians regarding vitamin B_12_ deficiency as a side effect of long-term metformin use. Although awareness levels were generally high, a substantial gap exists between understanding and practice. In one survey, 94% of physicians demonstrated sufficient awareness of B_12_ deficiency, but only 53% followed American Diabetes Association (ADA) recommendations.^33^ Furthermore, in a cross-sectional study conducted in primary care hospitals in Saudi Arabia, only 43% of physicians were aware of the need for annual B_12_ testing.^1^

The analysis of 4 studies^136^,^38^ that measured clinician awareness confirmed that many clinicians recognised the association between metformin and B_12_ deficiency, but a substantial proportion of clinicians underestimated the severity or failed to recall critical diagnostic thresholds. This was demonstrated in a study in which 94% of physicians recognised B_12_ deficiency as a potential consequence of metformin use, but more than half failed to identify the correct laboratory threshold for deficiency.^38^

Thus, the results of studies found through the scoping review would suggest that the problem is not that there is a lack of knowledge regarding the side effect, but that the clinical relevance of this side effect on the patients’ taking metformin is not well understood by practitioners. Nonetheless, the studies and literature available were limited and only related to clinicians working in the Middle East.

Objective 2—what are the existing screening and monitoring practices in primary care settings for early detection of vitamin B_12_ deficiency in patients on long-term metformin?

All the studies included in the scoping review that discussed screening recommended that this should happen in clinical practice. Some studies^1633 39^,^42^ allude to the guidance from organisations, such as the American Diabetes Association^43^ and the Medicines and Healthcare products Regulatory Agency^17^ as a source of guidance on screening. One study developed a novel screening tool for screening vitamin B_12_ deficiency in patients on long-term metformin and called the Metformin Usage Index (MUI).^44^

Nonetheless, the scoping review demonstrated that screening for vitamin B_12_ deficiency was suboptimal. The data from the review reveal that screening is often symptom-triggered rather than preventative (screening known high-risk patients). In several studies,^1 37 38^ fewer than 20% of at-risk patients received routine B_12_ testing, with only a small proportion of clinicians incorporating annual assessments into their practice. For example, a large-scale retrospective analysis revealed that only 4.4% of patients on long-term metformin had documented serum B_12_ levels.^36^ Similarly, a study reviewing over two million patient records reported B_12_ screening in just 2.6% of eligible cases.^39^

Additionally, a 2021 retrospective cohort analysis reviewed records of 13 489 insured patients with >1 year of metformin use.^34^ Less than half (44.9%) received a B_12_ test. On average, it took 990 days from metformin initiation for a test, and 1926 days for those who eventually tested positive.^42^

These figures reflect a pattern where B_12_ testing is symptom-driven rather than proactive, delaying diagnosis and potential intervention. This reactive approach may delay diagnosis, particularly in asymptomatic or elderly patients.

The review also identified that high-risk groups, such as elderly patients, those on higher doses or prolonged courses of metformin, vegans and individuals on proton pump inhibitors (all predisposed to vitamin B_12_ deficiency), are not consistently prioritised for screening.^45^ The results further suggest that patients over 75 years old were among the least likely to be tested despite having significantly lower B_12_ levels and higher risk of deficiency-related complications. For instance, T2DM patients over 75 were significantly less likely to be tested despite being at higher risk of deficiency.^37^

This is supported by a study of 592 patients with type 2 diabetes on metformin found 27.7% had confirmed vitamin B_12_ deficiency. The risk factors identified high metformin doses, age ≥80, and vegetarianism. The authors advocated routine screening as part of annual diabetes evaluations in primary care.^46^

While most clinicians recognised these risk factors, they were inconsistently acted on in practice. This misalignment highlights a critical gap in risk stratification and proactive care.

Objective 3—how is the evidence on clinician awareness and screening practices for vitamin B_12_ deficiency implemented in frontline clinical practice for patients with type 2 diabetes on long-term metformin?

The scoping review suggested that even when knowledge was present, its translation into routine practice was inconsistent. A few of the studies noted that although clinicians were aware of the risks and screening guidelines, practical implementation was lacking. The barriers cited included time constraints, lack of institutional prompts or tools, testing costs and competing clinical priorities.^140^,^42^ This gap indicates that knowledge alone is insufficient without structural and systemic support mechanisms.

To bridge the knowledge-practice gap, several studies called for system-wide changes.^1 39 41 42^ These included the introduction of electronic prompts, updated clinical guidelines, educational workshops and routine inclusion of B_12_ monitoring in diabetes management checklists. For example, in a quality improvement project, the introduction of a checklist prompt during T2DM reviews resulted in a substantial increase in B_12_ monitoring.^41^ The MHRA’s 2022^19^ drug safety update further encouraged regular monitoring in high-risk patients, aligning with ADA guidelines.^39^ In addition, the use of tools such as the MUI has been proposed for resource-limited settings to guide screening and supplementation decisions.^44^ Where such interventions were trialled, marked improvements in screening rates were observed, suggesting that clinician behaviour can be positively influenced through relatively simple structural changes.^44^

## Conclusions

Overall, the pool of evidence for clinician awareness of vitamin B_12_ deficiency in long-term metformin use is sparse. The studies that have been conducted are mainly based on Middle Eastern countries and no studies have been conducted in the UK. This represents a significant evidence gap within the UK primary care context, where most metformin prescribing and diabetes management occurs.

The scoping review demonstrated that our international primary care colleagues do not consistently screen for or manage vitamin B_12_ deficiency in long-term metformin users at the level recommended by emerging guidelines. Survey studies show that while awareness exists, only about half incorporate B_12_ checks into routine care.^1 38^

The scoping review confirmed that in the included studies, many patients are never tested, with testing rates declining despite guideline updates.^34 40^ This is despite proactive screening, especially in older patients and those with dietary risks, being essential. Supplementation is effective and inexpensive, yet missed diagnoses can lead to irreversible harm.

Recent findings support routine annual B_12_ screening for patients on long-term metformin, especially those on higher doses or with added risk factors. Few studies explicitly evaluated interventions aimed at improving clinician knowledge or screening behaviour, and there remains a notable absence of evidence examining how the MHRA 2022 Drug Safety Update^17^ has been implemented within UK primary care.

## Supplementary material

10.1136/bmjopen-2025-113829 online supplemental file 1

10.1136/bmjopen-2025-113829 online supplemental file 2

## Data Availability

All data relevant to the study are included in the article or uploaded as supplementary information.
