# Supplementary material for: Vitamin B12 deficiency in long-term metformin use and clinician awareness: a scoping review
Source: BMJ Open. 2026 Apr 20;16(4):e113829. doi: 10.1136/bmjopen-2025-113829 (PMC13110580; doi:10.1136/bmjopen-2025-113829)
Supplement: online supplemental file 1 [file bmjopen-16-4-s001.docx]

**Supplementary File 1. Full Electronic Search Strategies and Search Results**

This supplementary file details the electronic search strategies used for the scoping review, together with a summary of search results by database, reported in line with PRISMA-S guidance (Search Reporting Extension).

**Search period**

Database searches were conducted between 1^st^ August 2025 and 1^st^ November 2025. Searches included literature published from January 1990 onwards.

**Language limits**

All searches were limited to English-language publications.

**De-duplication**

All retrieved citations were imported into EndNote 21 (Clarivate). Automated de-duplication was undertaken, followed by manual review to remove remaining duplicates prior to screening.

**Full electronic search strategies**

**MEDLINE (PubMed)**

1. Vitamin B12.mp.
2. Metformin.mp.
3. Type 2 diabetes.mp.
4. Deficiency.mp.
5. Primary Care.mp.
6. Screening.mp. OR Early detection.mp.
7. 1 AND 2 AND 3 AND 4 AND 5 AND 6
8. Clinician.mp. OR Healthcare professional.mp. OR Doctor.mp. OR Nurse.mp. OR Pharmacist.mp.
9. Knowledge.mp. OR Awareness.mp.
10. 7 AND 8 AND 9

Limits applied: English language; January 1990–November 2025

**Embase**

1. Vitamin B12.mp.
2. Metformin.mp.
3. Type 2 diabetes.mp.
4. Deficiency.mp.
5. Primary Care.mp.
6. Screening.mp. OR Early detection.mp.
7. 1 AND 2 AND 3 AND 4 AND 5 AND 6
8. Clinician.mp. OR Healthcare professional.mp. OR Doctor.mp. OR Nurse.mp. OR Pharmacist.mp.
9. Knowledge.mp. OR Awareness.mp.
10. 7 AND 8 AND 9

Limits applied: English language; January 1990–November 2025

**CINAHL (EBSCO)**

1. Vitamin B12
2. Metformin
3. Type 2 diabetes
4. Deficiency
5. Primary Care
6. Screening OR Early detection
7. 1 AND 2 AND 3 AND 4 AND 5 AND 6
8. Clinician OR Healthcare professional OR Doctor OR Nurse OR Pharmacist
9. Knowledge OR Awareness
10. 7 AND 8 AND 9

Limits applied: English language; January 1990–November 2025

**Web of Science**

(Vitamin B12 AND Metformin AND Type 2 diabetes AND Deficiency AND Primary Care AND (Screening OR Early detection))
AND (Clinician OR Healthcare professional OR Doctor OR Nurse OR Pharmacist)
AND (Knowledge OR Awareness)

Limits applied: English language; January 1990–November 2025

**British Nursing Index (BNI)**

1. Vitamin B12
2. Metformin
3. Type 2 diabetes
4. Deficiency
5. Primary Care
6. Screening OR Early detection
7. 1 AND 2 AND 3 AND 4 AND 5 AND 6
8. Clinician OR Healthcare professional OR Doctor OR Nurse OR Pharmacist
9. Knowledge OR Awareness
10. 7 AND 8 AND 9

Limits applied: English language; January 1990–November 2025

**Cochrane Library**

Metformin AND Vitamin B12 AND Type 2 diabetes

Limits applied: English language; January 1990–November 2025

**Google Scholar**

Google Scholar was searched using combinations of the following terms: “metformin”, “vitamin B12 deficiency”, “type 2 diabetes”, “clinician awareness”, and “screening”. The first 200 results for each search combination were screened for relevance.

**Grey literature sources**

The following grey literature sources were searched using relevant keyword combinations: EThOS (British Library), DART-Europe E-theses Portal, and King’s College London Research Portal. Searches focused on clinician awareness, screening practices, and monitoring of vitamin B12 deficiency in patients receiving long-term metformin therapy.

**Search results summary**

| # | Searches | Results web of science | Results CINAHL | Results EMBASE | Results BNI | Results PubMed | Cochrane | Google scholar |
| --- | --- | --- | --- | --- | --- | --- | --- | --- |
| 1 | Vitamin B12 .mp. [mp=title, abstract, heading word, table of contents, key concepts, original title, tests & measures] | 60,332 | 9,704 | 67,196 | 412 | 37,504 | 2,599 | 1,490,000 |
| 2 | Metformin. mp. [mp=title, abstract, heading word, table of contents, key concepts, original title, tests & measures] | 70,567 | 13,281 | 99,044 | 661 | 3,263 | 14,088 | 876,000 |
| 3 | Type 2 diabetes. mp. [mp=title, abstract, heading word, table of contents, key concepts, original title, tests & measures] | 516,670 | 111,829 | 510, 714 | 10,356 | 24,773 | 65,679 | 4,160,000 |
| 4 | Deficiency. mp. [mp=title, abstract, heading word, table of contents, key concepts, original title, tests & measures] | 1,165,587 | 115,853 | 961,453 | 15,431 | 74,437 | 33,708 | 5,140,000 |
| 5 | Primary Care. mp. [mp=title, abstract, heading word, table of contents, key concepts, original title, tests & measures] | 740,614 | 139,542 | 907, 742 | 56,285 | 65,268 | 137,090 | 6,040,000 |
| 6 | Screening. mp. [mp=title, abstract, heading word, table of contents, key concepts, original title, tests & measures] | 5,060,416 | 221,029 | 1,501,014 | 37,792 | 636,475 | 108,431 | 4,970,000 |
| 7 | Early detection. mp. [mp=title, abstract, heading word, table of contents, key concepts, original title, tests & measures] | 537,380 | 280,932 | 283,589 | 3,236 | 61,656 | 17,880 | 6,280,000 |
| 8 | 1 and 2 and 3 and 4 and 5 and 6 or 7 | 9 | 12 | 14 | 8 | 11 | 14 | 84 |
| 9 | Clinician. mp. [mp=title, abstract, heading word, table of contents, key concepts, original title, tests & measures] | 83 | 132,036 | 163,278 | 21,794 | 34,607 | 32,893 | 4,580,000 |
| 10 | Healthcare professional. mp. [mp=title, abstract, heading word, table of contents, key concepts, original title, tests & measures] | 80,126 | 50,960 | 66,901 | 25,331 | 82,082 | 5,884 | 5,780,000 |
| 11 | Doctor. mp. [mp=title, abstract, heading word, table of contents, key concepts, original title, tests & measures] | 152,918 | 91,687 | 268,036 | 32,702 | 78,565 | 20,406 | 5,180,000 |
| 12 | Nurse. mp. [mp=title, abstract, heading word, table of contents, key concepts, original title, tests & measures] | 414,272 | 533,820 | 458,637 | 324,732 | 47,234 | 59,314 | 7,290,000 |
| 13 | Pharmacist. mp. [mp=title, abstract, heading word, table of contents, key concepts, original title, tests & measures] | 54,386 | 31,163 | 125,180 | 3,851 | 5,467 | 6,424 | 1,360,000 |
| 14 | Knowledge. mp. [mp=title, abstract, heading word, table of contents, key concepts, original title, tests & measures] | 56 | 317,802 | 1,337,558 | 60,426 | 111,087 | 57,245 | 7,330,000 |
| 15 | Awareness. mp. [mp=title, abstract, heading word, table of contents, key concepts, original title, tests & measures] | 418,098 | 134,813 | 358,848 | 20,046 | 34,623 | 21,626 | 5,910,000 |
| 16 | 9 or 10 or 11 or 12 or 13 and 14 or 15 | 104,2665 | 134, 813 | 12,138 | 621,000 | 51, 255 | 4,234 | 2,640,000 |
| 17 | 1 and 2 and 3 and 4 and 16 | 6 | 1 | 1 | 0 | 6 | 0 | 1 |
| 18 | Limit 17 to English | 6 | 1 (duplicate) | 1 | 0 | 6 | 0 | 1 |
