# Supplementary material for: Vitamin B12 deficiency in long-term metformin use and clinician awareness: a scoping review
Source: BMJ Open. 2026 Apr 20;16(4):e113829. doi: 10.1136/bmjopen-2025-113829 (PMC13110580; doi:10.1136/bmjopen-2025-113829)
Supplement: online supplemental file 2 [file bmjopen-16-4-s002.docx]

| Name of study/article | Author | Year of publication | Origin/country | Aims/purpose | Population and sample size | Methodology/methods | Outcomes and details of these | Key findings that relate to scoping review |
| --- | --- | --- | --- | --- | --- | --- | --- | --- |
| American Diabetes Association. Standards of Care Diabetes-2025 | Editor-Khan, S. | 2024 | USA | The American Diabetes Association (ADA) “Standards of Care in Diabetes,”, serves as a comprehensive resource to clinicians, researchers, policy makers, and other stakeholders. It outlines key elements of diabetes care, sets treatment goals, and provides tools to assess care quality, all aimed at improving diabetes care and outcomes across diverse populations. | N/A | The methodology for data collection in the ADA Standards of Care in Diabetes—2025 document is based on a systematic review of evidence and an assessment of the benefits and risks of various diabetes care options. Here are the key aspects of the methodology | Metformin and Vitamin B12 Deficiency  Long-term metformin use is associated with decreased vitamin B12 levels.  The risk of vitamin B12 deficiency (<200 pg/mL or <150 pmol/L) is notably higher in patients on metformin for 4–5 years.  Patients on higher doses (≥1,500 mg/day) or longer treatment durations have an increased risk​.  Screening Recommendations:  Annual monitoring of vitamin B12 levels is advised for patients who:  Have been on metformin for more than four years.  Have additional risk factors, such as a vegan diet or a history of gastric/small bowel surgery.  Show symptoms of deficiency, including anaemia or peripheral neuropathy​. Clinical Practice Integration-  The document emphasises periodic vitamin B12 assessment as an integral part of diabetes management for long-term metformin users.  It suggests testing vitamin B12 levels when deficiency symptoms appear, particularly in those with neuropathy or anaemia​ | Objective 1 – No relevant points discussed  Objective 2- In primary care settings, vitamin B12 screening and monitoring practices include: Annual Monitoring: For patients on metformin for over four years or those at risk due to dietary patterns (e.g., vegan diets) or previous gastric/small bowel surgery, annual vitamin B12 monitoring is advised. Symptom-Based Testing: Serum vitamin B12 levels should be checked if deficiency is suspected, especially in patients presenting with anaemia or peripheral neuropathy​.  Objective 3- No relevant points discussed. |
| Physicians’ Perception About Use of Vitamin B12 in the Treatment or Prevention  of Diabetic Neuropathy: A Cross-sectional Survey in Saudi Arabia | Khaled K. Aldossari, Sameer Al-Ghamdi, Jamaan Alzahrani, Maram S. Al Turki, Mai  Almuhareb, Ziad G. Alanazi, Ziyad M. Alshahrani and Jencia Wong | 2022 | Saudia Arabia | The aim of this study is to examine the awareness  of Saudi physicians of vitamin B12’s inability to treat Diabetic Neuropathy and to assess how commonly it is prescribed  as a treatment for this condition. | A total of 412 physicians with a  minimum of three years of experience | A cross-sectional study, conducted between May and November of 2019, in several  primary healthcare centers in different cities of Saudi Arabia.  Physicians answered a three-part questionnaire on their demographic information,  prescribing behaviour, and knowledge of the relationship between vitamin B12 or vitamin  B complex and diabetic neuropathy. | The study found that only 42% of the physicians believed that vitamin B12 supplementation  did not prevent diabetic neuropathy, while only 52.7% found it to be an ineffective treatment  for this condition. 43% of respondents were aware  that vitamin B12 levels should be tested annually. The findings of this study indicate a tendency of unnecessarily prescribing vitamin  B12 supplementation for the prevention or treatment of diabetic neuropathy as well as a lack of  knowledge on the matter among doctors in primary care hospitals in Saudi Arabia. | Objective 1- The paper concluded that this study has several implications. Most importantly, it  shows that there is confusion and misinformation among  physicians in Saudi Arabia about the treatment of diabetic  neuropathy. Additionally, it shows that physicians are not always  updated with new literature, especially in regard to vitamin  B12.  Objective 2- The study suggested that there  be updated awareness campaigns on vitamin B12, how often  its deficiency should be tested  Furthermore, the study concluded that there  should be programs to ensure diabetic patients, especially  those taking metformin, are tested for vitamin B12 deficiency  every year, and only given supplement prescriptions  based on those annual tests.  Objective 3- No relevant points discussed. |
| Vitamin B12 Deficiency in Patients with Diabetes on Metformin:  Arab Countries | Jwaher Haji Alhaji | 2022 | Saudi Arabia | The paper aimed to conduct a preliminary review of the  literature (observational and interventional studies) to determine the relationship between  metformin use for treatment of T2DM and vitamin B12 deficiency (as measured by vitamin  B12 concentration in the blood), and to identify possible complications | The authors searched for all relevant studies published in English prior to 2020 on Web of Knowledge  databases: PubMed, Web of Science, and Google Scholar, using the following search  terms “metformin”, “vitamin B12”, “neuropathy”, “diabetes” and Middle Eastern countries. | A literature review of observational and interventional studies. | The author identified 7257 records. Out of those, 11 studies were included in the  review. The review indicated an association  between taking metformin and B12 deficiency in patients with T2DM in Arab countries. The vitamin B12  deficiency was found to be negatively associated with the dose and duration of metformin therapy.  The physician’s knowledge of current ADA recommendations regarding supplementation with  and screening of the vitamin B12 level for T2DM patients on metformin was also found to have an effect. | Objective 1- One study in the review found that many physicians lacked awareness of current ADA recommendations regarding B12 supplementation and screening in type 2 diabetes patients on metformin.  Objective 2- The paper overall concludes that routine vitamin B12 level testing is not widely implemented in Arab healthcare settings, and many patients on long-term metformin do not receive B12 supplementation unless deficiency symptoms appear.  Objective 3- No relevant points were discussed. |
| Evaluating Physician Knowledge, Attitudes, and  Practices in Screening and Supplementation for  Vitamin B12 Deficiency in Type 2 Diabetes  Patients Treated with Metformin | Samaher Z AlSaad, Razan K AlHadlaq, Enas Fahad Alaraik, Abdullah O Alnomany,  Haytham I AlSaif , Turky H Almigbal, Mohammed A Batais, Abdullah A Alrasheed | 2024 | Saudi Arabia | This study aims to assess the knowledge, attitude, and practice among Riyadh-based physicians regarding Vitamin B12 supplementation and deficiency screening, to inform the implementation of clinical practice guidelines for the screening, treatment, and prevention of Vitamin B12 deficiency in T2DM patients on metformin therapy | Physicians of diverse specialties—including residents, specialists, fellows, and consultants—working in various government hospitals and primary care centers in Riyadh, Saudi Arabia. The survey was conducted between January 2019 and January 2020. Exclusion criteria for the study encompassed physicians employed in private hospitals and private primary care centers, as well as those who were unable to participate due to various reasons or who declined to complete the survey. | A survey was administered to physicians across various specialties in government hospitals and primary care centers in Riyadh, Saudi Arabia, from January 2019 to January 2020. The survey assessed their knowledge, attitudes, and practices concerning Vitamin B12 deficiency screening and supplementation | There were 402 participating physicians in the study, of those- 94.0% (378 respondents) demonstrated sufficient knowledge about Vitamin B12 deficiency.  Physicians with more extended years of experience showed significantly better knowledge about Vitamin B12 screening and supplementation.  While the study showed most physicians were knowledgeable about Vitamin B12 deficiency and supplementation, a substantial gap in translating this knowledge into practice was observed. The study recommended a critical need for institutional oversight to ensure adherence to American Diabetes Association (ADA) guidelines for Vitamin B12 screening and supplementation in T2DM patients on long-term metformin therapy | Objective 1- 94% of respondents demonstrated sufficient knowledge about vitamin B12 deficiency. However, 23% of respondents underestimated vitamin B12 as a side effect of metformin. However, 83% of respondents recognised age as a risk factor for developing vitamin B12 deficiency on metformin. Furthermore, 56% of respondents were not able to correctly identify the lab cut off level for vitamin B12 deficiency. 52% of respondents recognised that a higher dose of metformin increases the risk of vitamin b12 deficiency.  Objective 2- 49.8% reported routinely screening for Vitamin B12 deficiency in symptomatic patients, and 40.5% indicated they would repeat screening if previous levels were low. A significant majority (70.1%) performed routine patient screening, including detailed history taking, physical examination, and laboratory testing. The most used laboratory test for assessing Vitamin B12 deficiency was serum vitamin B12 levels, as reported by 84.1% of the physicians. 78% of respondents felt that vitamin B12 deficiency in metformin use should be assessed at each visit.  Objective 3- No relevant points were discussed. |
| Vitamin B12 deficiency and the knowledge and practice  of physicians regarding screening for vitamin B12  deficiency among type 2 diabetic patients on metformin  in selected hospitals in Riyadh, Saudi Arabia | Amal N. Alshammari, Rafay Iqbal, Iqra Peer Baksh | 2019 | Saudi Arabia | This study aimed to find  the prevalence of vitamin B12 deficiency in metformin‑treated  T2DM patients in selected Ministry of Health (MoH) hospitals  in Riyadh, Saudi Arabia, and determine the knowledge and  perception of physicians on vitamin B12 screening, to be able  to make a recommendation for screening, as suggested by ADA  and the European Association for the study of diabetes. | Part 1 included 363 T2DM patients on metformin Part 2 included100 physicians who care for patients with T2DM and on metformin | Part 1 A cross‑sectional study was conducted at the diabetic clinics  at four selected MoH hospitals in Riyadh in which data was collected from the medical record using a data extraction form.  Part 2 A self-administered questionnaire was distributed to all clinicians conducting diabetic clinics. | This study found that only 4.4% of the study population  had available serum vitamin B12 levels, which means that serum  levels of vitamin B12 were not routinely requested.  This study also highlighted that a large  number of the included physicians were not aware or updated with  the current recommendations regarding vitamin  B12 supplementation and vitamin B12 screening for T2DM  patients on metformin. | Objective 1-A large percentage of physicians are not  aware of the current recommendations regarding  vitamin B12 supplementation and screening.  Objective 2- States that only 4.4% of relevant patients were screened for Vitamin B12 deficiency but gives no recommendations regarding screening.  Objective 3- Explores there is a lack of awareness and recommends increased awareness is required but does not explore reasons or factors for lack of awareness |
| Assessment of the knowledge and practice of Jordanian family  medicine practitioners regarding vitamin B12 screening for type 2  diabetes mellitus patients on metformin | Maysaa Alwadi , Rawan Badaineh , Tareq Alwedyan , Esraa Gogazeh , Ola A Badir | 2023 | Jordan | To evaluate  the knowledge and practice of family physicians among diabetic patients on metformin, and to evaluate the factors that enhance and prevent vitamin  B12 screening | 147 family physicians participated in the study | A validated questionnaire was designed and distributed online for family medicine practitioners between June and September  2022. A scoring system was used to calculate the knowledge, practice, and total scores | The  results reveal a high total knowledge score among participants about vitamin B12 manifestations and screening in T2DM patients. However, only half of the participants follow the ADA recommendations routinely (53.06) %. The total practice score is low as most participants (87.7%) answer only 9 of  18 questions correctly. Moreover, the results show that those who follow ADA recommendations routinely have better knowledge and practice scores  (p-value 0.00406) | Objective 1- The study showed while 86.39% of physicians recognised B12 deficiency as a side effect of metformin, only 12.24% considered the risk to be high.  About 53.06% of respondents followed ADA recommendations on B12 screening, but knowledge did not always translate into practice.  Objective 2- 97.96% of physicians had ordered B12 tests for diabetic patients on metformin, but only 58.86% did so for asymptomatic patients.  Periodic monitoring was not common—only 19.73% ordered routine B12 tests, while most tested only when patients were symptomatic or on high doses.  Objective 3-  Barriers to screening included lack of tools (90.48%), high test cost (61.22%), and patient refusal (50.34%)​. The study suggests workshops, updated clinical guidelines, and better lab accessibility to improve B12 monitoring and treatment in frontline clinical practice​ |
| Trends in Vitamin B12 Level Testing in Patients on  Metformin From 2000 to 2020 | Debarati Bhanja, Areeba Zain, Camille Moeckel, Abdul Waheed | 2024 | USA | This study examines trends over time in the  use of vitamin B12 level testing among patients on metformin | Patients treated with metformin from 2000 to 2020 were identified using Rx Concept Unique  Identifier codes. 4,203,020 patient records were analysed | retrospective trend analysis used data from TriNetX, a real-world, longitudinal clinical  database. Patients treated with metformin from 2000 to 2020 were identified using Rx Concept Unique  Identifier codes. The number of patients who underwent vitamin B12 level testing at any time after 1  month from metformin initiation was tabulated. Patients were grouped by the year of B12 level testing.  Trends in B12 level testing were assessed using the Jonckheere-Terpstra statistical test | This retrospective cohort study found that use of vitamin B12 level testing in patients on metformin has  significantly declined over the past 20 years, thereby impacting screening and early detection of B12 deficiency  in clinical practice. | Objective 1- study was not designed to explore causes for  down-trending B12 testing rates; however, they hypothesised that B12 testing may be underutilised for several  reasons. However, a  knowledge gap may exist among providers regarding the American Diabetes Association B12 monitoring  recommendations, or low prioritisation of B12 testing may exist compared to other diabetes guidelines  Objective 2- No relevant points discussed  Objective 3- The study concludes that collaborative efforts between health care practitioners and researchers will be crucial for further  research on metformin-associated B12 deficiencies. |
| Metformin use and vitamin B12 deficiency:  New MHRA guidance | Sarah Davies | 2022 | UK | This article is a brief review of metformin-associated B12 deficiency and its  implications for practice, including screening, treatment and monitoring | n/a | n/a | This article concluded that vitamin B12 deficiency is now viewed as a common side effect of metformin therapy. That people taking metformin, especially if high-dose or long-term, and particularly if  they have other risk factors for deficiency, should have their B12 levels checked periodically. That many patients with deficiency  will require intramuscular  B12 replacement for the  duration of their metformin  treatment, although a trial  of oral replacement initially  may be worthwhile. | Objective 1- No relevant points discussed.  Objective 2- The MHRA advises screening for B12 deficiency in patients who:  Take high-dose or long-term metformin.  Have risk factors like older age, gastrointestinal conditions (Crohn’s, gastrectomy), or vegan diets.  Screening should be symptom-based, particularly for neuropathy, anaemia, and cognitive impairment.  Annual monitoring of B12 levels is recommended in at-risk patients, representing a shift in clinical practice.  Objective 3 – No relevant points discussed. |
| Should we screen diabetic patients using biguanides  for megaloblastic anaemia? | Kalitsa Filioussi, Stefanos Bonovas, Thomas Katsaros | 2003 | Australia | The aim of this observational study  was to estimate the prevalence of megaloblastic  anaemia among ambulatory  diabetic patients on long term treatment  with biguanide | 600 patients with type 2 diabetes treated with biguanides (phenformin or metformin) | The study screened patients with type 2 diabetes treated with biguanides (phenformin or metformin) for a mean  of 11.8 years (SD: 3.6 years) with complete blood counts, red cell indices and red cell morphology. If this showed  macrocytosis, the study measured total serum vitamin B12 and antiparietal cells antibodies (APCA). | Megaloblastic anaemia was discovered in  54 out of the 600 patients on long term  biguanides with a prevalence of low  serum total vitamin B12 levels of 9% | Objective 1- No relevant points discussed.  Objective 2- Annual screening  for megaloblastic anaemia in patients on  long term treatment with biguanides is  worthwhile because the complication is  remediable and does not necessitate withdrawal  of the drug.  Objective 3- No relevant points discussed. |
| Vitamin B12 screening in metformin-treated diabetics in primary care: were elderly patients less likely to be tested? | Yacov Fogelman , Eliezer Kitai, Gari Blumberg, Avivit Golan-Cohen, Micha Rapoport, Eli Carmeli | 2016 | Israel | The objective of this study was to examine the current practice and clinical determinants of vitamin B12 testing in metformin treated T2DM patients. | Participants were patients, who were T2DM, patients who were newly prescribed metformin from 2008 to 2013. Patients were randomly divided into two subgroups: referred for a vitamin B12 blood test, and did not receive a referral | Of them 5131 T2DM patients (the study population) had not received metformin in the previous years and began taking it during the study period and continued doing so for at least 6 consecutive months without interrupting treatment for more than 29 days.  After searching their electronic medical records, they were assigned, to either group A: patients referred for vitamin B12 blood test by their family physicians and group B who were not referred. | 5131 patients began taking metformin during the study period. Of these 2332 (44.5 %) had vitamin B12 tested.  A significant positive association (*p* < .05) was found between vitamin B12 testing and insulin treatment, retinopathy, neuropathy and hypertension. Vitamin B12 in elderly (>75 years) patients was significantly lower (*p* < .01). Insulin treatment, hypertension, and chronic diabetic complications in metformin treated T2DM patients are associated with higher rates of vitamin B12 testing. T2DM patients 75 years and above were less likely to be tested for B12 deficiency.  The ramifications of these facts is that too many cases of B12 deficiency which can be easily treated are being missed, most keenly in elderly diabetics treated with metformin. And since only part of the damage can be alleviated, an earlier diagnosis would lead to a better outcome. We call upon guideline producers to take this important subject into account and upon all primary care physicians to be aware. | Objective 1- No relevant points discussed  Objective 2- Calls on guidelines to promote screening of vitamin B12 deficiency screening in T2DM on metformin.  Objective 3- no relevant points dsicussed. |
| Prevalence of Vitamin B12–Monitoring among Patients Receiving Metformin in a Public Hospital | Ronak Ghiya,  and Maya Fayfman | 2022 | USA | To better understand acceptance and application of these guidelines, we undertook an assessment of such monitoring within a single hospital center serving the low-income population in Atlanta | 2,336,914 T2DM patients who were taking metformin. | Data was collected from a single hospital center using electronic medical records (EMR) for all outpatients for whom metformin was prescribed either as a single agent or in combination therapy during January 1 - December 31, 2021 and then measure Vitamin B12 prescribing among primary care patients and patients followed by endocrinology | Of the total 2,336,914 patients notes reviewed, 30,803 (0.01%) met the eligibility criteria. Of these, only 816 patients (2.6%) had vitamin B12 levels ordered and measured in outpatient clinics. In primary care clinics, vitamin B12 levels ordered and measured were at 7.5% (674 patients). Among two cohorts who were prescribed metformin, there were suboptimal adherence to the ADA’s recommendation for regular monitoring of Vitamin B12 levels. | Objective 1- The study alluded that there was sub-optimal adherence to the ADA screening recommendation but failed to elicit a reason for this.  Objective 2- The study suggests the ADA screening should be followed by clinicians.  Objective 3- The study emphasises that approaches are required to improve adherence to the monitoring regime and suggest that approaches should be developed for this, but does not describe these approaches. |
| Discovering metformin-induced vitamin B12 deficiency in patients with type 2 diabetes in primary care | Laura Herbert, Alicia Ribar, Sheryl Mitchell, & Cynthia Phillips, | 2021 | USA | The purpose of this project was to determine  whether a check list intervention in a primary care clinic,  that follows the guidelines suggested by the ADA, would  cause practice improvement in discovering metformin-  induced vitamin B12 deficiency. The project aimed to  identify vitamin B12 deficiency in patients with type 2 diabetes who were taking metformin | The project was conducted in an estab- lished internal medicine primary care practice and included three physicians and two nurse practitioners who all have more than 3 years in clinical practice. The project sample population consisted of data of adults aged 18 years and older (N = 1,479) seen in this clinic during the project period with the diagnosis of type 2 diabetes mellitus | This was a quasi-experimental project of preintervention and postintervention design using a checklist containing important measures of diabetes control. The project sample population consisted of data of adults with type 2 diabetes aged 18 years and older who were prescribed metformin in the previous year at the primary care practice | There was a significant improvement in monitoring vitamin B12 levels and discovery of low vitamin B12 levels. These data show that the number of B12 levels checked increased from 23 during the pre-intervention to 155 during the intervention. | Objective 1- No relevant points discussed.  Objective 2- The paper recommends following the ADA recommendations for screening for vitamin B12 deficiency whilst on metformin.  Objective 3- The project team discussed how introducing a prompt on the checklist for a T2DM review increased vitamin B12 monitoring. |
| Evaluation of vitamin B12 monitoring in patients on metformin in urban ambulatory care settings | Stacy L. Longo, Jessica M. Ryan, Kelsey B. Sheenan, Debra J. Reid, Michael P. Conley,  Carla J. Bouwmeester | 2019 | USA | The primary objective was to assess the occurrence of vitamin B12 level monitoring within the past 5 years in patients with type 2 diabetes treated with metformin, and to determine if these levels were normal, low, or deficient. | Patients aged 18 years and older with a diagnosis of type 2 diabetes and an active prescription for metformin | This was a retrospective chart review of adult patients with type 2 diabetes on metformin doses ≥ 1000 mg for ≥ 6 months at five Federally Qualified Health Centers (FQHC) and one Program of All-Inclusive Care for the Elderly (PACE). Charts were reviewed for occurrence of monitoring vitamin B12 levels in the past 5 years | 322 patients included and 25% had a vitamin B12 level measured in the previous five years. Among the patients with a vitamin B12 level, 87.7% were within the normal range. Each greater year of age was associated with a 5% increased odds of vitamin B12 monitoring. | Objective 1- No relevant points discussed  Objective 2- The study showed  most of the patients who were monitored had normal vitamin B12 levels, which may warrant extending the monitoring time. They concluded that their finding may also support monitoring patients who have additional risk factors for vitamin B12 deficiency such as concurrent medication use with other vitamin B12 lowering agents or clinical symptoms of deficiency such as peripheral neuropathy. They suggested future studies are needed to determine appropriate frequency of monitoring.  Objective 3- No relevant points discussed. |
| Assessment of vitamin B12 deficiency  and B12 screening trends for patients on  metformin: a retrospective cohort  case review | Darby Martin, Jeet Thaker, Maria Shreve, Lois Lamerato,  Kartazyna Budzynska | 2021 | USA | This study investigated the use of vitamin  B12 testing in a large cohort of patients on metformin  and assesses appropriateness and benefits of screening  recommendations for vitamin B12 deficiency | A diverse, adult, insured population of  patients who had more than 1 year of metformin use  between 1 January 2010 and 1 October 2016 and who  filled at least two consecutive prescriptions of metformin | A retrospective cohort study that included insured  adult patients who had more than 1 year of metformin use  between 1 January 2010 and 1 October 2016 and who had  filled at least two consecutive prescriptions of metformin  to establish compliance. The comparison group was not  exposed to metformin. The primary outcome was incidence  of B12 deficiency diagnosed in patients on metformin.  Secondary outcome was occurrence of B12 testing in the  patient population on metformin. Records dated through  31 December 2018 were analysed. | 13 489 patients on metformin, 6051 (44.9%)  were tested for vitamin B12 deficiency, of which 202 (3.3%)  tested positive (vs 2.2% of comparisons). Average time  to test was 990 days. Average time to test positive for  deficiency was 1926 days. Factors associated with testing  were linked to sex (female, 47.8%), older age (62.79%  in patients over 80 years old), race (48.98% white) and  causes of malabsorption (7.11%). Multivariable logistic  regression showed older age as the only factor associated  with vitamin B12 deficiency. individuals on metformin  were tested for vitamin B12 deficiency more frequently  if they were elderly, had a malabsorption disorder, or they  were taking proton pump inhibitors. African-American  ethnicity approached significance as a protective factor  for B12 deficiency. | Objective 1- Study conclusion is that clinicians should be cognisant of the increased risk of vitamin B12 deficiency in select populations. No measurement of clinician awareness recorded.  Objective 2- Screening should be undertaken inn high risk groups such as prolonged metfomin use > 65yrs old and other co-prescribing such as PPIs as well as consideration of racial background.  Objective 3- No relevant points discussed. |
| Increasing Vitamin B12  Screening Among  Patients With Type 2  Diabetes on Long-Term  Metformin Therapy | Elijah P. Mays, Jr. | 2021 | USA | This was report on a Quality Improvement (QI) project in improving screening of Vitamin B12 deficiency in patients on Metformin > 2 years using the ADA guidelines | Patients who attended this clinic with T2DM and was on metformin > 2 years. | A notes audit was undertaken for 1 year prior to the changes in the guidelines to establish a baseline of screening for vitamin B12. A training program and initiatives were put in place for relevant staff working in the clinic to improve screening rates. The rate of Vitamin B12 screening was measured monthly for 4 months afterwards. | The baseline audit shows that 68% of patients on metformin > 2 years had not been screened for vitamin B12 deficiency (32% had been screened. After the training program screening rates initially improved by 11% in the first month and 24% in the second month. These rates dropped significantly in the last 2 months due to the start of the COVID pandemic and change in operational practice due to social distancing. | Objective 1- Does not explore specifically clinician awareness but looks at screening rates pre and post QI project.  Objective 2- Promotes the screening regime as described by the ADA, but does not explore the effectiveness of screening tools etc. Explores the uptake of the screening tool by clinicians, working in a clinic with T2DM patients.  Objective 3- As part of the QI project it explores some of the barriers and challenges in increasing clinician awareness, such as time, communication and competing priorities for clinicians, such as the COVID pandemic. |
| Vitamin B12 Deficiency in Patients Taking  Metformin: Pathogenesis and Recommendations | Noor ul Huda Ramzan , Khadija Shahjahan , Rubaid A. Dhillon , Nimra Tul Ain Khan ,  Muhammad Bilal Hashmat , Mian Uman Anwer , Dawood Ahmed , Fazila Afzal ,  Muhammad Mashhood Tahir , Ayesha Muzaffar | 2024 | Pakistan | This review aims to synthesize current knowledge on the pathogenesis, prevalence, clinical  implications, and management of metformin-induced vitamin B12 deficiency | N/A | No methodology for conducting the review was described in the paper. | The review showed that clinical evidence indicates that metformin  use can lead to vitamin B12 deficiency.  Prevention and  treatment of vitamin B12 deficiency are simple and effective; therefore, clinicians should periodically  monitor vitamin B12 levels in patients prescribed metformin.  Additionally, more research is needed to determine whether specific  subgroups, such as race or gender, are more prone to developing vitamin B12 deficiency. This will help tailor  guidelines and lead to better patient outcomes for those who are most susceptible | Objective 1- No relevant points discussed.  Objective 2- Discussed the MHRA 2022 screening guidelines. A screening tool (Metformin Usage Index created by Shivaprasad et al 2020) is suggested in table 2 by the authors as an effective screening tool in practice.  Objective 3-No relevant points discussed. |
| Medicines and Healthcare products Regulatory Agency (MHRA)- Drug Safety Update Vol 15 issue 11 | Medicines and Healthcare products Regulatory Agency (MHRA) | 2022 | UK | To disseminate new information and guidance about the risk of vitamin B12 deficiency in long term metformin use and when to appropriately screen for this. | n/a | n/a | The MHRA Drug Safety Update (June 2022) highlights that vitamin B12 deficiency is a common side effect of metformin, affecting up to 1 in 10 patients, particularly those on higher doses or long-term treatment. The MHRA now recommends testing vitamin B12 levels in patients with symptoms suggestive of deficiency, such as megaloblastic anaemia, neuropathy, cognitive impairment, or fatigue. Additionally, periodic B12 monitoring should be considered for high-risk individuals, including those with low baseline B12 levels, gastrointestinal disorders (e.g., Crohn’s disease, gastric surgery), vegan diets, or concomitant use of medications that impair absorption (e.g., PPIs, colchicine). Clinicians are advised to continue metformin therapy while correcting B12 deficiency with supplementation and to follow established clinical guidelines for B12 deficiency management | Objective 1- No relevant points discussed  Objective 2- The MHRA now advises periodic vitamin B12 monitoring for at-risk patients, particularly those with:  Lower baseline B12 levels, Gastrointestinal disorders (e.g., Crohn’s disease, gastric surgery)  Strict vegan/vegetarian diets, Concomitant use of medications affecting B12 absorption (e.g., proton pump inhibitors).  Testing is particularly recommended for patients presenting with anaemia, neuropathy, cognitive impairment, or other B12 deficiency symptoms​.  Objective 3- The MHRA update resulted in changes to metformin product labelling, instructing prescribers to consider B12 deficiency risk and monitor accordingly. |
| Metformin Usage Index and assessment of vitamin B12 deficiency  among metformin and non‑metformin users with type 2 diabetes  mellitus | Channabasappa Shivaprasad · Kolla Gautham· Barure Ramdas· Kolli S. Gopaldatta·  Krishnamurthy Nishchitha. | 2020 | India | The study aimed to evaluate the combined effect of both dose and duration of metformin therapy on vitamin  B12 levels in patients with type 2 diabetes mellitus. | 2887 patients with T2D who are categorised into metformin and non-metformin users | Between January 2018 and November 2019- the study calculated the  “Metformin Usage Index” (MUI) which was defined as the product of the dose of metformin (mg) used and its duration  divided by 1000. Vitamin B12 levels were compared between the two groups, and its association with MUI was assessed  using correlation and multistep logistic regression analyses. | Vitamin B12 levels < 200 pg/ml and between 200 and 300 pg/ml were noted among 24.5% and 34.5% metformin  users, respectively; this was significantly higher than among non-metformin users (17.3% and 22.6%, respectively). The MUI can be employed as a risk assessment tool for evaluation of vitamin B12 deficiency in patients with  T2D. | Objective 1- No relevant points discussed  Objective 2- The paper does not directly outline existing screening practices for vitamin B12 deficiency in primary care settings but suggests that routine monitoring is not widely implemented. It highlights the potential use of the Metformin Usage Index (MUI) as a tool to identify high-risk patients, recommending that patients with an MUI >5 should be screened for B12 deficiency. The study also notes that in resource-poor settings, an MUI >5 could serve as a threshold for initiating vitamin B12 supplementation without the need for costly screening.  Objective 3- No relevant points discussed |
| Person-Centered Primary Care and Type 2 Diabetes:  Beyond Blood Glucose Control | Kimberly K. Trout,  William F. McCool,  Carol J. Homko | 2019 | USA | This article reviews the current literature with  regard to the initial evaluation of individuals who are diagnosed with diabetes, and what is entailed in comprehensive continuing management  of care. A person-centered interprofessional approach to care of the person with diabetes is presented by the authors | N/a | N/a | Knowledge of current research identified and discussed effective care practices for individuals with diabetes. | Objective 1- No relevant points discussed  Objective 2- This article mentions that prolonged use of metformin can result in vitamin B12 deficiency, and periodic measurement of vitamin B12 levels should be considered for those who are on prolonged therapy.  Objective 3- No relevant points discussed |
| Should people treated  with metformin be  screened for vitamin  B12 deficiency? | Claire Holt | 2012 | UK | The article discusses the health and cost  implications of screening for vitamin B12 deficiency in people with  diabetes taking metformin, alongside the 10 principles outlined in  the World Health Organization publication *Principles and Practice*  *of Screening for Disease: Public Health Papers No. 34* (Wilson and  Jungner, 1968) | N/a | N/a | The article suggests that metformin  reduces the absorption of vitamin B12 and  increases the risk of deficiency. Approximately  one in 10 adults with type 2 diabetes taking  metformin develop vitamin B12 deficiency within  4.3 years of commencing therapy. Screening those at risk of deficiency might  prevent pathological changes developing and  harm occurring; however, formal screening  programmes need to be supported by sufficient  evidence and justified in health economic terms. | Objective 1- No relevant points discussed  Objective 2- In 2012 when article was published there was no national screening program. However, given the risks of health deterioration with vitamin B12 deficiency healthcare professionals should at least remember to check Vitamin b12 levels in anyone with diabetes and peripheral neuropathy.  Objective 3- No relevant points discussed |
| Vitamin B12 deficiency among patients with  diabetes mellitus: is routine screening and  supplementation justified? | Davis Kibirigeand Raymond Mwebaze | 2017 | Uganda | This review article offers a current perspective on the physiological roles  of vitamin B12, proposed pathophysiological mechanisms of vitamin B12 deficiency, screening for vitamin B12  deficiency and vitamin B12 supplementation among patients with diabetes mellitus. | N/a | N/a | Clinical and biochemical vitamin B12 deficiency is highly  prevalent among patients with both types 1 and 2 DM.  Future large and well-designed studies on screening for  vitamin B12 deficiency, vitamin B12 supplementation and  optimal supplementation  are warranted to help guide formulation  of guidelines. Annual screening  for vitamin B12 deficiency using more sensitive methods  like serum homocysteine and methylmalonic acid concentrations  should be adopted. | Objective 1- No relevant points discussed  Objective 2- When this article was written in 2013 there was no published guidelines for screening. They summarised, that the patient should be screened for vitamin B12 prior to commencing metformin and on an annual basis for elderly patients, on long term metformin (> 3-4yrs), suffering from peripheral neuropathy or on high dose metformin (>2g/day)  Objective 3- No relevant points discussed |
| Metformin Use and  Vitamin B12 Deficiency:  Managing the Risks | Paula Peacock | 2019 | USA | This article reviews the correlation between metformin use and vitamin b12 deficiency. It further considers if guidelines for vitamin b12 screening should be consiudered | N/a | N/a | The article confirms there is a link between prolonged metformin use and vitamin b12 deficiency and that screening is required. Though suggest further research is required to look at the frequency and extent of screening required. It suggest by undertaking screening and treating those patients appropriately an improvement in health outcomes would be beneficial to healthcare providers and patients. | Objective 1- No relevant points discussed  Objective 2-It discusses how at the time that the ADA guidelines have only been released, which called for the assessment of risk for Vitamin B12 deficiency. The article also discusses the cost of screening and treating these patients (from an American Healthcare point of view vs the cost of the outcomes if not treated). It concludes screening would be beneficial but offers nor further guidance.  Objective 3- No relevant points discussed |
| Determinants of vitamin B12 deficiency in patients with type-2 diabetes mellitus - A primary-care retrospective cohort study | Andrew Kien Han Wee, Rehena Sultana | 2023 | Singapore | The aim of this study is to investigate the determinants of B12 deficiency in community-dwelling patients with T2DM, with the aim of profiling patients most in need of B12-deficiency screening | 592 primary-care patients with T2DM, recruited from 2008 to 2011 | Vitamin B12 deficiency was evaluated using a retrospective cross-sectional cohort of 592 primary-care patients with T2DM, recruited from 2008 to 2011 from a Polyclinic in Singapore. | B12 deficiency (serum B12 < 150 pmol/L) was present in 164 (27.7%) patients and was associated with a higher "metformin daily dose". Independent of the duration of T2DM, 29.3% of the B12-deficient patients needed > 1 screening test before the detection of B12 deficiency.  Primary-care screening for B12 deficiency should be part of the annual laboratory review of patients with T2DM regardless of the duration of T2DM -especially when they are prescribed ≥ 1.5 g/day of metformin; ≥ 80 years old; vegetarian; and not prescribed B12 supplementation. Concurrent evaluation for associated folate (vitamin B9) deficiency is essential when addressing T2DM-associated B12 deficiencies | Objective 1- No relevant points discussed  Objective 2- The study recommended that Primary-care screening for B12 deficiency should be part of the annual laboratory review of patients with T2DM regardless of the duration of T2DM —especially when they are prescribed ≥ 1.5 g/day of metformin; ≥ 80 years old; vegetarian; and not prescribed B12 supplementation.  Objective 3- No relevant points discussed |
