# Supplementary material for: Vitamin B12 deficiency in long-term metformin use and clinician awareness: a scoping review
Source: BMJ Open. 2026 Apr 20;16(4):e113829. doi: 10.1136/bmjopen-2025-113829 (PMC13110580; doi:10.1136/bmjopen-2025-113829)
Supplement: Supplementary file 3 [file bmjopen-2025-113829.reviewer_comments.pdf]

PEER REVIEW HISTORY

BMJ Open publishes all reviews undertaken for accepted manuscripts. Reviewers are asked to complete a checklist review form and are provided with free text boxes to elaborate on their assessment. These free text comments are reproduced below.

ARTICLE DETAILS

Title (Provisional)

Scoping review: Vitamin B12 deficiency in long term metformin use and clinician awareness

Authors

Parsonage, Ian; Wainwright, David; Barratt, Julian

VERSION 1 - REVIEW

**Reviewer**	**1**
**Name**	**Ferraro, Simona**
**Affiliation**	**Vittore Buzzi Children's Hospital**
**Date**	**09-Dec-2025**
**COI**	**None**

-Spell out all acronyms the first time. see T2DM in the first lines of the introduction.

- About no consensus on the diagnostic cut-off. The main issue is that harmonized cut-off values have been recommended (see UK NICE guidelines for B12 deficiency over 16ys , in this guideline metformin use is quoted as well known risk factor).

Anyway laboratory assays measuring B12 levels suffer for a poor agreement between results and accordingly one sigle cut-off is not reliable, but cut-off values related to the different assays should be considered.

This issue is important and it has to be quoted, since clinicians should be aware of the fact that testing the same patients in different laboratories may obtain different results.

This reference may be of aid to clarify this issue in the discussion. "Assessing the harmonization of current total vitamin B12 measurement methods: relevance and implications. Clin Chem Lab Med. 2025 Aug 20. doi: 10.1515/cclm-2025-0939". Another paper has tried for clarity to convert the threshold from on assay to another doi: 10.21037/jlpm-25-49.

Reviewer **2**

Name **Shetty, Ranjitha**

Affiliation **Kasturba Medical College Manipal, Department of Community Medicine**

Date **15-Jan-2026**

COI **None**

Abstract: The abstract was written appropriately aligned with the objectives and content of the manuscript.

However, the authors must undertake substantial revisions in the following areas of the manuscript to enhance its overall strength and ensure greater clarity for readers.

Introduction:

1. The introduction could be concise; it is relatively lengthy and includes background information that extends beyond what is necessary for the current manuscript. The authors may consider this section by focusing more closely on information that directly supports the study objectives, which would improve clarity and enhance the overall flow of the paper.

2. Including a clear definition of clinical awareness in the introduction would strengthen the manuscript by improving conceptual clarity.

3. Clearly articulating the research gap, along with the study rationale / aim, at the end of the introduction would improve the logical flow of the manuscript.

Methodology:

1. The methodology section requires clearer organization. Structuring the methods in a systematic manner would improve readability and transparency.

2. Aligning the methodology with the PCC (Population, Concept, Context) framework may strengthen conceptual clarity and ensure consistency with the stated review objectives.

3. The detailed search strategy provided at the end of the document could be given an appropriate title and explicitly referred to in the main text where the search process is described.

4. Generic explanations like grey literature may be omitted if they do not directly contribute to methodological rigor.

5. Full-text screening was conducted by a single reviewer, and involving an additional independent reviewer would strengthen methodological rigor and reduce potential selection bias.

Results

1. Although the Results section includes a heading on appraisal and synthesis of results, a formal critical appraisal does not appear to have been conducted; clarifying this and avoiding overlap in terminology would improve consistency and transparency.

2. The abbreviation ADA should be written in full at its first appearance in the manuscript to ensure clarity for readers.

3. In the Results section, Figure 2 (visual map of themes generated from the analysis using a weighted list of key themes) is mentioned but not adequately described. Highlighting the most important findings would strengthen the results presentation.

Discussion:

The manuscript uses the terms clinician knowledge and clinician awareness in a similar context; standardizing terminology or explicitly defining any conceptual distinction would enhance clarity.

Conclusion:

The conclusion states that no studies explored barriers and facilitators; however, barriers are reported under Objective 3 in the Discussion section, and this should be addressed for alignment.

VERSION 1 - AUTHOR RESPONSE

Reviewer 1 comments
Use of acronyms All acronyms (e.g. type 2 diabetes mellitus) are now spelled out at first use.
Diagnostic thresholds and assay variability The Discussion has been revised to clarify that, while guideline-recommended diagnostic thresholds exist (including NICE guidance), uncertainty in practice relates primarily to variability between laboratory assays rather than a lack of guidance. This issue has been explicitly discussed, with reference to recent work on assay harmonisation and implications for clinical interpretation.
Reviewer 2 comments
Introduction The Introduction has been streamlined to focus on information directly supporting the study objectives. A clear operational definition of clinician awareness has been added, and the research gap and study rationale are now explicitly articulated at the end of the section.
Methodology The Methods section has been reorganised for clarity and aligned with the PCC (Population, Concept, Context) framework. Eligibility criteria and

search terms are now presented in tabular format. Generic methodological explanations have been reduced where not essential, and limitations relating to single-reviewer full-text screening are transparently acknowledged.

Results Terminology relating to appraisal and synthesis has been clarified to reflect scoping review methodology, with explicit confirmation that no formal critical appraisal was undertaken. Abbreviations (e.g. American Diabetes Association) are spelled out at first use. Figure 2 has been more fully described in the Results text, highlighting the dominant and less frequently represented themes identified across the literature.

Discussion and conclusion alignment Terminology relating to clinician awareness and knowledge has been standardised for consistency. The Conclusion has been revised to accurately reflect findings relating to barriers and facilitators, ensuring alignment with the Results and Discussion.

We believe these revisions have substantially strengthened the manuscript and addressed all editorial and reviewer concerns. We would like to thank the Editor and reviewers again for their time and valuable input, and we hope the revised manuscript is now suitable for publication in BMJ Open.

Yours sincerely,

Ian Parsonage (on behalf of all authors)
